# Effects of Inclusion of *Schizochytrium* spp. and Forage-to-Concentrate Ratios on Goats’ Milk Quality and Oxidative Status

**DOI:** 10.3390/foods10061322

**Published:** 2021-06-08

**Authors:** Alexandros Mavrommatis, Kyriaki Sotirakoglou, Charalampos Kamilaris, Eleni Tsiplakou

**Affiliations:** 1 Laboratory of Nutritional Physiology and Feeding, Department of Animal Science, School of Animal Biosciences, Agricultural University of Athens, Iera Odos 75, GR-11855 Athens, Greece; mavrommatis@aua.gr; 2 Laboratory of Mathematics and Statistics, Department of Natural Resources and Agricultural Engineering, School of Environment and Agricultural Engineering, Agricultural University of Athens, Iera Odos 75, GR-11855 Athens, Greece; sotirakoglou@aua.gr; 3 School of Geosciences, University of Edinburgh, Drummond Street, Edinburgh EH8 9XP, UK; harry.kamilaris@sruc.ac.uk

**Keywords:** microalgae, fatty acid, antioxidant, grains, forage, starch, MDA, DHA, TAC, rumen

## Abstract

Although the dietary inclusion level of polyunsaturated fatty acids (PUFA) and the forage: concentrate (F:C) ratio affect milk quality, their interaction has not been broadly studied. To address such gaps and limitations a two-phase trial using twenty-two dairy goats was carried out. During the first phase, both groups (20 HF *n* = 11; high forage and 20 HG *n* = 11; high grain) were supplemented with 20 g *Schizochytrium* spp./goat/day. The 20 HF group consumed a diet with F:C ratio 60:40 and the 20 HG-diet consisted of F:C = 40:60. In the second phase, the supplementation level of *Schizochytrium* spp. was increased to 40 g/day/goat while the F:C ratio between the two groups were remained identical (40 HF *n* = 11; high forage and 40 HG *n* = 11; high grain). Neither the *Schizochytrium* spp. supplementation levels (20 vs. 40) nor the F:C ratio (60:40 vs. 40:60) affected milk performance. The high microalgae level (40 g) in combination with high grain diet (40 HG) modified the proportions of docosahexaenoic acid (DHA), docosapentaenoic acid (DPA), and conjugated linoleic acid (CLA) and the ω3/ω6 ratio in milk, to a beneficial manner according to human health recommendation guidelines. However, the highest inclusion level of *Schizochytrium* spp. (40 g) and foremost in combination with the high grain diets (40 HG) induced an oxidative response as observed by the increased protein carbonyls (CP) and malondialdehyde (MDA) levels in milk and blood plasma indicating severe limitations for a long-term, on-farm application. In conclusion, the supplementation with 20 g *Schizochytrium* spp. and high forage diet (60:40) appears to be an ideal formula to enrich dairy products with essential biomolecules for human health without adversely affect milk oxidative stability.

## 1. Introduction

In the Western diet, the average intake of the health-beneficial ω3 long-chain polyunsaturated fatty acids (LCPUFA) such as the eicosapentaenoic acid (EPA) and docosahexaenoic acid (DHA) is below the recommended level, raising interest to enrich foods with ω3 LCPUFA [1]. In this context, several feedstuffs and novel feed additives enriched with bioactive fatty acids have been tested in ruminant nutrition aiming to alter milk fatty acid profile [2]. Amongst these efforts, the inclusion of microalgae in animals’ diets appears to be the most sustainable and vegetable-friendly strategy to improve polyunsaturated fatty acid (PUFA) milk content [3,4,5,6,7,8]. More specifically, *Schizochytrium* spp., a unicellular eukaryote belonging to Thraustochytriaceae family, appears to be a genus that walks the line between marine fungi and microalgae, exploiting the structure and properties of both kingdoms. Its heterotrophic cultivation depicts a promising perspective since it is feasible to produce highly valuable nutrients by utilizing low-cost substrates such as organic wastes without the dependence on sunlight [9]. Notably, the supplementation with 20, 40, and 60 g *Schizochytrium* spp./day in goats’ diet increased milk docosapentaenoic acid (DPA), DHA, and conjugated linoleic acid (CLA) content, and ω3/ω6 ratio [7]. However, high inclusion levels of *Schizochytrium* spp. in goats’ diet with a moderate forage to concentrate (F:C = 50:50), ratio reduced the abundance of cellulolytic microbes and caused milk fat depression [10,11].

The F:C ratio, is also a keystone factor that alters rumen fermentation and milk chemical composition [12]. A decrease in milk fat and PUFA content was found, when the crushed linseeds- (rich in PUFA) fed cows shifted from high to a low forage diet [13]. Although a meta-analysis study in cows pointed out that marine oils compared to linseeds cause a sharp decrease in their milk fat content [14], we speculated that alterations in the F:C ratio with various inclusions levels of PUFA-rich microalgae simultaneously, could further modulate goats milk composition and fatty acid profile.

However, the high propensity of PUFA to oxidation could severely affect animals and products oxidative balance as well [15]. Notably, the inclusion of 40 and 60 g *Schizochytrium* spp./day in goats’ diet induced a cascade of pro-oxidant incidences in both blood and milk resulting in a compromised oxidative status [16]. Furthermore, changes in the F:C ratio could regulate ruminants’ oxidative status as well. Interestingly, in goats, a high grain diet increased the lipopolysaccharides (LPS) level in their ruminal fluid, resulting in a low-grade pro-inflammatory response and oxidative stress [17]. Specifically, the mRNA levels and activities of glutathione peroxidase (GSH-Px), catalase (CAT), and superoxide dismutase (SOD) were decreased in goat’s liver, while malondialdehyde (MDA) concentration was upsurged [17]. Additionally, the high starch level increases the degradation of exogenous antioxidants compounds such as vitamins A, E, and the carotenoids within the rumen resulting in a lower antioxidant ability of the organism [18,19].

Even though both lipids supplementation and F:C ratio alterations portray well-documented dietary interventions in ruminants, scarce information exists about their interactions on milk composition and its oxidative stability. Taking into consideration the aforementioned and our preliminary results from previous trials on *Schizochytrium* spp. in goats diet [7,10,11,16], the objective of this study was to evaluate the impact of two inclusion levels of *Schizochytrium* spp. (20 g and 40 g/day) and two forage to concentrate ratios (60/40 and 40/60) (a) on milk performance and chemical composition, (b) on the fatty acid profile of blood plasma and milk, and on (b) GSH-Px, CAT, SOD, glutathione reductase (GR), glutathione transferase (GST) activities in blood plasma and SOD, GR, CAT, and lactoperoxidase (LPO) activities in milk and (c) total antioxidant capacity and oxidative stress indicators (MDA and protein carbonyls (PCs) in both blood plasma and milk of dairy goats.

## 2. Materials and Methods

### 2.1. Diets and Experimental Design

The study was conducted with respect to the guidelines of the European Union Directive on the defense of animals used for scientific purposes (EU 63/2010; Council of the European Union 2010). Twenty-two crossbred dairy goats (Alpine × Local (Greek) breeds) at early lactation (70 ± 10 days in milk), were separated into two homogenous groups (*n* = 11 per group) according to their age (3–4 years old), body weight (BW; 50.6 ± 6.1 kg), and (4 fat corrected %) milk yield (FCM_4%_). The experimental trial was divided into two phases (two dietary groups each), which lasted 8 weeks each, with the first 2 weeks being an adaptation period. During the first phase, each goat of both groups (20 HF; high forage and 20 HG; high grain) was supplemented with 20 g *Schizochytrium* spp./day. The F:C ratio of the 20 HF group was 60% forages (alfalfa hay and wheat straw) and 40% concentrate while that of 20 HG was 40% forages (alfalfa hay and wheat straw) and 60% concentrates (Table 1). In the second phase, the supplementation level of *Schizochytrium* spp. was increased to 40 g/day/goat while the F:C ratio between the two groups were remained identical (40 HF; high forage and 40 HG; high grain) (Table 1). *Schizochytrium* spp. is a commercial product traded as DHAgold by the DSM feed industry (DSM Nutritional Products, Marousi, Greece). The *Schizochytrium* spp. were added into concentrate mix aiming to provide 20 and 40 g/goat/day in both high forage (1 Kg concentrate/goat/day; 20 g/*Schizochytrium* spp./Kg in 20 HF and 40 g/*Schizochytrium* spp./Kg in 40 HF) and high grain (1.3 Kg concentrate/goat/day; 15.4 g/*Schizochytrium* spp./Kg in 20 HF and 30.7 g/*Schizochytrium* spp./Kg in 40 HF) diets (Table 1; Table S1). The rations were designed to be isocaloric and isonitrogenous (Table 1; Table S1). The alfalfa hay, wheat straw and concentrates samples were analyzed for organic matter (OM; Official Method 7.009), dry matter (DM; Official Method 7.007), and crude protein (CP; Official Method 7.016) according to the Association of Official Analytical Chemists (1984) using a Kjeldahl Distillation System (FOSS Kjeltec 8400, Demark). Neutral detergent fiber (NDF) and acid detergent fiber (ADF) expressed exclusive of residual ash according to the method of Van Soest using an ANKOM 2000 Fiber Analyzer (New York, NY, USA) as described by Tsiplakou et al. [20] (Table S2). Non-fibrous carbohydrates were calculated based on the equation described by Cannas et al., [21]. Feed samples were also analyzed for fatty acids profile according to the method of O’Fallon et al., [22] (Table 2). The forages (alfalfa hay and wheat straw) were provided separately from the concentrates. Animals were fed on a group basis, considering their average energy and nutritional requirements in order for the experimental design to represent the typical commercial farm feeding management and the results having practical implications for small ruminants. The available feeding space was higher than the one recommended for adult housed goats (0.33 m per animal) considering to favor simultaneous access and lower competitive interactions at the feeder among animals. Forage was provided with the concentrate in two equal portions after milking. Diet consumption was being recorded on daily basis.

### 2.2. Sample Collection

The goats were milked two times per day (08:00 and 17:00) with a milking machine. Individual milk samples (*n* = 264; 11 goats/group × 4 groups (2 experimental phases of 2 dietary groups each) × 6 sampling times) were collected on the 7th, 14th, 21th, 28th, 35th, and 42nd experimental day of each experimental phase and used for milk chemical composition. While milk samples obtained from 21th and 42nd experimental days (*n* = 88; 11 goats/group × 4 groups × 2 sampling times) of each experimental phase were used for fatty acid profile and oxidative status analyses. Milk yield was recorded at the same experimental days (7th, 14th, 21st, 28th, 35th, and 42nd) taking into account the two milked quantities, while each of the aforementioned individual milk samples was performed by the mixture of 5% of the milk volume obtained by the two milkings aiming to ensure the highest reliability.

Individual blood samples (*n* = 88) were also taken on the 21st and 42nd experimental days (of each experimental phase) from the jugular vein of each goat after the milking prior to access on feeds. Approximately, 10 mL of whole blood were immediately transferred to heparin-containing tubes (170 units heparin; BD Vacutainer, Plymouth, UK) and stored in an icebox (Thomas Scientific, Swedesboro, NJ, USA) until their transfer to the Laboratory of Nutritional Physiology and Feeding. Then, the blood samples were centrifuged (SL16R, Thermo Fisher Scientific, Waltham, MA, USA) at 2500 rpm for 15 min at 4 °C to separate plasma from the cells.

Milk samples for chemical composition were analyzed on the collection day, while milk and blood plasma samples were stored at −80 °C, prior to fatty acids and oxidative status analyses.

### 2.3. Milk Chemical Composition

Chemical composition (fat, protein, and lactose) was analyzed using an IR spectrometer (MilkoScan 133; FOSS, Hillerød, Demark) after proper validation by Kjeldahl [23] and Gerber [24] methods. Fat corrected- (FCM_4%_) and energy corrected- (ECM) milk yield was calculated using the following formulas:

Fat corrected milk (FCM) in 4% based on the Equation (1)

FCM_4%_ = (0.40 + 0.15 × F) × M (1)

where F = fat content (%) and M = milk yield in kg [3].

Energy corrected milk (ECM) yield based on the Equation (2)

ECM = (milk yield × 0.327) + (fat yield × 12.95) + (protein yield × 7.2)(2)

### 2.4. Milk and Blood Fatty Acid Analysis

The plasma fatty acid analysis was carried out with the method of Bondia-Pons et al. [25] as previously described by Tsiplakou et al. [26]. Milk samples were analyzed for fatty acid according to the method of Nourooz-Zadeh and Appelqvist [27] as described by Tsiplakou et al. [28]. FA profile was performed using an Agilent 6890 N gas chromatograph equipped with an HP-88 capillary column (60 m × 0.25 mm i.d. with 0.20 µm film thickness, Agilent). Information about the temperature program and standard used are available by Mavrommatis and Tsiplakou [7]. The groups of FA were defined as follow:

Short-Chain Saturated Fatty Acids (SCFA) = C_6:0_ + C_8:0_ + C_10:0_ + C_11:0_, (3)

Medium-Chain Saturated Fatty Acids (MCFA) = C_12:0_ + C_13:0_ + C_14:0_ + C_15:0_ + C_16:0_, (4)

Long-Chain Saturated Fatty Acids (LCFA) = C_17:0_ + C_18:0_ + C_20:0_, (5)

Mono-Unsaturated Fatty Acids (MUFA) = C_14:1_ + C_15:1_ + C_16:1_ + C_17:1_ + _cis-9_ C_18:1_ + _trans-11_ C_18:1_ + _trans_ C_18:1_, (6)

Poly-Unsaturated Fatty Acids (PUFA) = _cis-9, trans-11_ C_18:2_ + C_18:2n-6c_ + C_18:2n-6t_ + C_18:3n-3_ + C_18:3n-6_ + C_20:3n-3_, (7)

Saturated Fatty Acids (SFA) = SCFA + MCFA + LCFA,(8)

Unsaturated Fatty Acids (UFA) = PUFA + MUFA,(9)

Saturated/Unsaturated (S/U) = (SCFA + MCFA + LCFA)/(PUFA + MUFA),(10)

Atherogenic index (AI) = (C_12:0_ + 4 x C_14:0_ + C_16:0_)/(PUFA + MUFA), (11)

Thrombogenic index (TI) = (C_14:0_ + C_16:0_ + C_18:0_)/(0.5 × MUFA) + (0.5 × n-6 PUFA) + (3 × ω3 PUFA) + (ω3 PUFA/ω6 PUFA), (12)

Health promoting index (HPI) = (ω6 PUFA + ω3 PUFA + MUFA)/(C_12:0_ + 4 × C_14:0_ + C_16:0_). (13)

### 2.5. Antioxidant Enzymes Activities and Oxidative Status Indicators

The assays for antioxidant enzyme activities, oxidative stress indicators, and the total antioxidant capacity were performed using a UV/Vis spectrophotometer (GENESYS 180, Thermo Fisher Scientific, Waltham, MA, USA) as previously described [29]. The GSTs activities were recorded by monitoring the conjunction of GSH to 1-chloro-2,4-dinitrobenzene (CDNT) at 340 nm. CAT activity was performed using a commercial spectrophotometric kit (Catalase Assay Kit; CAT100, Sigma-Aldrich, St. Louis, MO, USA). GSH-Px activity was assayed according to Paglia and Valentine [30]. GR activity was performed by measuring the reduction of oxidized glutathione (GSSG) to reduce glutathione in presence of nicotinamide adenine dinucleotide phosphate (NADPH) at 340 nm. SOD activity was recorded by monitoring the inhibition of cytochrome c oxidation at 550 nm. LPO activity in milk was performed by monitoring the oxidation of 2,2′-Azino-bis (3-ethylbenzthiazoline-6-sulfonic acid) ABTS in presence of hydrogen peroxide at 340 nm. MDA was measured according to Nielsen et al. [31] with some modifications described by Mavrommatis et al. [32]. The protein carbonyls (PC) were assayed according to the method of Patsoukis et al. [33]. The ABTS [34,35] and the ferric reducing ability of plasma (FRAP) [36] assays were used to assess the total antioxidant capacity.

### 2.6. Statistics

**Figure 1:**
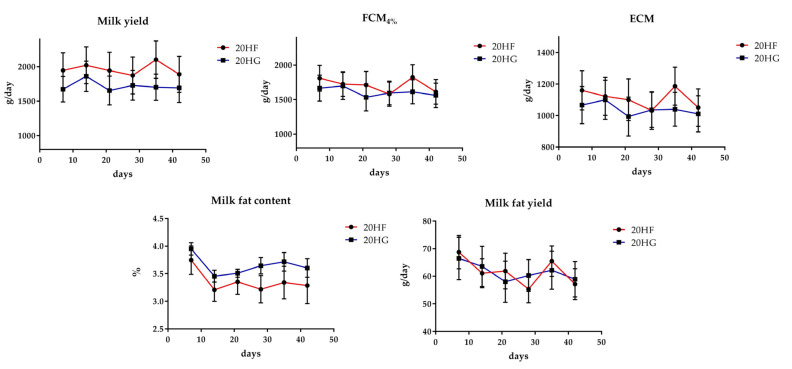
Mean milk performance of goats fed diets with 20 g *Schizochytrium* spp. and two different forage to concentrate ratios (20 HF; red line and 20 HG; blue line) throughout the experimental period. Error bars represent the standard error of the means (SEM). 20 HF: 20 g *Schizochytrium* spp. and high forage diet (60:40); 20 HG: 20 g *Schizochytrium* spp. and high grain diet (40:60).

**Figure 2:**
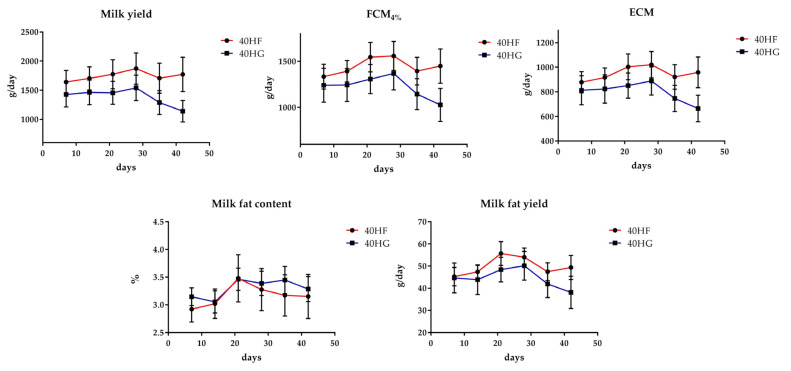
Mean milk performance of goats fed diets with 40 g *Schizochytrium* spp. and two different forage to concentrate ratios (40 HF; red line and 40 HG; blue line) throughout the experimental period. Error bars represent the standard error of the means (SEM). 40 HF: 40 g *Schizochytrium* spp. and high forage diet (60:40); 40 HG: 40 g *Schizochytrium* spp. and high grain diet (40:60).

Dataset was evaluated in SPSS.IBM software (v 20.0) and the results are depicted as mean ± standard error of means (SEM). The milk yield and body weight of each experimental phase were analyzed separately to avoid the effect of the lactation stage. The effect of dietary treatment between two groups in both experimental phases was assessed by performing a GLM for repeated measures analysis of variance. The dietary treatments (D) (D = 20 HF and 20 HG for phase 1 and 40 HF and 40 HG for phase 2) were defined as the fixed factor and the sampling time (S) as the repeated measure, while their interactions (D × S) were also assessed, according to the following model:

(14) $$Y_{ijkl}=\mu +D_{i}+S_{j}+A_{k}+{\left(D\times S\right)}_{ij}+e_{ijkl}$$

where is $Y_{ijkl}$ the dependent variable, μ the overall mean, $D_{i}$ the effect of dietary treatment (*i* = 2; 20 HF and 20 HG for phase 1 and 40 HF and 40 HG for phase 2), $S_{j}$ the effect of sampling time (*j* = 6; 7th, 14th, 21st, 28th, 35th, and 42nd experimental day), $A_{k}$ the animal’s random effect, ${\left(D\times S\right)}_{ij}$ the interaction between dietary treatments and sampling time, and $e_{ijkl}$ the residual errors. A total of 132 observations (11 goats × 2 dietary groups × 6 sampling times) were emerged for each experimental phase. Posthoc analysis was applied when appropriate using Tukey’s multiple range test. For all tests, the significance level was set at *p* = 0.05. Simplifying the visualization of these results, GraphPad Prism 6.0 (2012) depicted interleaved bars (Figure 1 and Figure 2 and Figure S1A,B).

Discriminant analyses were also performed (variables were entered independent together) on fatty acids and oxidative status pooled data (both in blood plasma (A) and milk (B)) to establish those variables capable of distinguishing and classifying samples amongst the four dietary groups (20 HF, 20 HG, 40 HF, and 40 HG). Wilk’s lambda (λ) criterion was used for assessing discriminant functions [37]. Sixteen and forty-seven variables for blood and milk fatty acid profile and nine and six for blood plasma and milk oxidative status were entered to create four models to distinguish the eighty-eight samples of each case (4 groups × 11 goats/group × 2 sampling time). Moreover, Pearson correlations were performed on milk fatty acid profile aiming to unveil significant correlations between individual fatty acids.

Blood and milk fatty acid profiles and oxidative status of blood plasma and milk were analyzed using a GLM for three-way repeated-measures ANOVA, considering the forage to concentrate ratio (F/C) (60/40, 40/60) as the between-subjects factor and microalgae level (A) (20 g, 40 g) and sampling time (S) (21st, 42nd experimental day) as within-subjects factors and the interactions among them according to the model:

(15) $$Y_{ijklm}=\mu +{\left(F/C\right)}_{i}+A_{j}+S_{k}+G_{l}+{\left(F/C\times A\right)}_{ij}+{\left(F/C\times S\right)}_{ik}+{\left(A\times S\right)}_{jk}+{\left(F/C\times A\times S\right)}_{ijk}+e_{ijklm}$$

where $Y_{ijklm}$ is the dependent variable, μ the overall mean, ${\left(F/C\right)}_{i}$ the effect of forage to concentrate ratio (*i* = 2; 60/40 and 40/60), $A_{j}\;$ the effect of microalgae level (*j* = 2; 20 g and 40 g),$\;S_{k}$(*k =* 2; 21st and 42nd experimental day), $G_{l}$ the animal’s random effect, ${\left(F/C\times A\right)}_{ij},\;{\left(F/C\times S\right)}_{ik},\;{\left(A\times S\right)}_{jk},\;{\left(F/C\times A\times S\right)}_{ijk}$ the two-way and three-way interactions between the aforementioned factors of the experiment and $e_{ijklm}$ the residual errors. Posthoc analysis was applied when appropriate using Tukey’s multiple range test. For all tests, the significance level was set at *p* = 0.05.

## 3. Results

### 3.1. Feed Intake and Body Weight

The mean wheat straw intake was decreased by 34% and 50% in the 20 HF and 40 HF groups respectively (Table 3). The mean concentrate intake was also decreased in both 40 HF and 40 HG groups by 16%. These changes also decreased the microalgae intake since they have been supplemented into the concentrates (40 HF; 33.7 g and 40 HG; 33.2 g vs. the planned of 40 g; Table 3). However, the planned F:C ratios and NDF to starch proportion were not considerably modified (Table 3). The mean body weight (BW) of goats did not differ among the dietary groups in both experimental phases (Figure S1A,B, Tables S3 and S4).

### 3.2. Milk Performance and Chemical Composition

In Figure 1 and Figure 2 are depicted the milk performance and fat content of each one of the two experimental phases respectively. Milk yield, energy- and fat-corrected milk yield, and milk chemical composition did not differ amongst the dietary groups.

### 3.3. Blood Plasma and Milk Fatty Acid Profile

**Figure 3:**
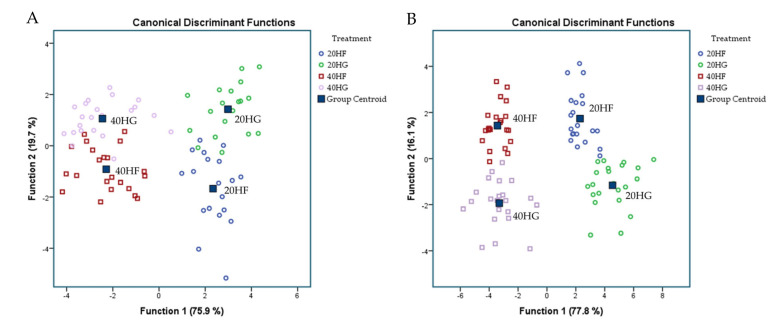
Discriminant plots separating (**A**) the four dietary treatments (20 HF; blue, 20 HG; green, 40 HF; red, and 40 HG; pink) according to pooled data of two sampling time (21st and 42nd experimental day) on the blood plasma fatty acid profile and (**B**) the four dietary treatments (20 HF; blue, 20 HG; green, 40 HF; red, and 40 HG; pink) according to pooled data of two sampling time (21st and 42nd experimental day) on the milk fatty acid profile. 20 HF: 20 g *Schizochytrium* spp. and high forage diet (60:40); 20 HG: 20 g *Schizochytrium* spp. and high grain diet (40:60); 40 HF: 40 g *Schizochytrium* spp. and high forage diet (60:40); 40 HG: 40 g *Schizochytrium* spp. and high grain diet (40:60).

Figure 3A depicts a discriminant plot of blood plasma fatty acid profile of the four dietary treatments (20 HF; blue, 20 HG; green, 40 HF; red, and 40 HG; pink) throughout the experimental period. The proportions of the samples that were correctly classified were 91.6%. Wilks’ lambda was observed at 0.034 for Function 1 (*p* < 0.001) and 0.264 for Function 2 (*p* < 0.001), while the proportions of C_22:2 n6_, C_16:1_, C_18:2 n6 cis_, C_18:1 trans_, C_18:3 n3_, and C_22:6 n3_ in blood plasma were the variables that contributed the most based on a step wise method. The four dietary treatments are clearly classified apart excepting a few minors overlapping between the same microalgae level groups. However, within Function 1, which describes 75.9% of the model, the level of microalgae possesses the dominant role. Figure 3B depicts the second discriminant plot of milk fatty acid profile of the four dietary treatments (20 HF; blue, 20 HG; green, 40 HF; red, and 40 HG; pink) throughout the experimental period. The proportions of the samples that were correctly classified were 98.9%. Wilks’ lambda was observed at 0.010 for Function 1 (*p* < 0.001) and 0.137 for Function 2 (*p* < 0.001), while the proportions of C_24:1_, C_15:0_, C_18:2 n6 cis_, C_17:1_, C_20:4 n6_, C_16:0_, and C_6:0_ in milk were the variables that contributed the most based on a step wise method. The four dietary treatments are clearly classified apart without observing any overlapping in the observations, however, within Function 1, which describes 77.8% of the model, the level of microalgae possesses the dominant role as well.

Table 4 depicts the blood fatty acid profile. Myristic acid (C_14:0_) was significantly (*p* < 0.001) increased in the blood of high microalgae-fed goats (40 HF and 40 HG); the same trend (*p* < 0.01) was found in the 42nd experimental day compared to 21th (Table 4). These changes unveiled a significant interaction (*p* < 0.001) between microalgae level and sampling time (Table 4). Palmitic (C_16:0_) and palmitoleic acids (C_16:1 n-7_) were significantly (*p* < 0.05 and *p* < 0.001 respectively) increased in high microalgae-fed goats (40 HF and 40 HG); while in palmitic acid, a significant interaction (*p* < 0.01) between sampling time and F:C ratio was found (Table 4). Stearic acid (C_18:0_) was significantly (*p* < 0.001) decreased in high microalgae-fed goats (40 HF and 40 HG; Table 4). Vaccenic acid (VA) in blood plasma (C_18:1 *trans*-11_) tended to increase (*p* = 0.072) in high grain diets (20 HG and 40 HG) and significantly increased (*p* < 0.001) in high microalgae-fed goats (40 HF and 40 HG; Table 4). Oleic acid (C_18:1 cis-9_) was significantly (*p* < 0.001) decreased in high microalgae-fed goats (40 HF and 40 HG); the same trend (*p* < 0.001) was found in the 42th experimental day compared to 21th (Table 4). These fluctuations resulted in significant (*p* < 0.05) interactions amongst sampling time and F:C ratio and between all investigated factors (*p* < 0.05). Linoleic acid (C_18:2 n-6 cis_) was significantly (*p* < 0.001) decreased in high microalgae-fed goats (40 HF and 40 HG), while linolenic acid (C_18:3 n-3_) was increased in high forage diets (20 HF and 40 HF; Table 4). Dihomo-γ-linolenic acid (C_20:3 n-6_), Eicosatrienoic acid (C_20:3n3_), docosadienoic acid (C_22:2 n-6_), Docosapentaenoic acid (C_22:5 n-6_), and Docosahexaenoic acid (C_22:6 n-3_) were significantly (*p* < 0.001) increased in high microalgae-fed goats (40 HF and 40 HG; Table 4).

The mean individual fatty acids (FA), grouped FA, FA health indices, and Δ-9 desaturase indices of milk are presented in Table 5. Long-chain fatty acids (LCFA) were significantly (*p* < 0.01) decreased by 41% in high (40 HF and 40 HG) compared to low microalgae-fed goats (Table 5). Monounsaturated fatty acids (MUFA) were significantly (*p* < 0.05) increased by 13% in high forage diets (20 HF and 40 HF). Polyunsaturated fatty acids (PUFA) and unsaturated fatty acids (UFA) were significantly increased by 27% (*p* < 0.001) and 6% (*p* < 0.05), while the proportion of saturated fatty acids (SFA) were decreased (*p* < 0.05) in high (40 HF and 40 HG) compared to low microalgae-fed goats (Table 5). The ω6 fatty acids were increased (*p* < 0.05) in high grain diets (20 HG and 40 HG), while ω3 showed a significant upsurge in high microalgae-fed goats (40 HF and 40 HG) resulting in changes in ω6/ω3 ratio (Table 5).

Atherogenic index (AI) was significantly (*p* < 0.05) decreased (2.37 vs. 1.79) in high grain diets (20 HG and 40 HG), while the opposite was found in high (40 HF and 40 HG) compared to low microalgae-fed goats (Table 5). Thrombogenic index (TI) was considerably (*p* < 0.001) decreased (1.62 vs. 1.41), while the health-promoting index (HPI) of milk fatty acid profile was increased (*p* < 0.001) in high (40 HF and 40 HG) compared to low microalgae-fed goats (Table 5). The high inclusion level of *Schizochytrium* spp. was found to increase the activity of Δ-9 desaturases as was indirectly indicated by the proportions of C_16:1_/C_16:0_ and C_18:1_/C_18:0_ (Table 5).

Individually, pentadecanoic acid (C_15:0_) in milk was significantly (*p* < 0.001) decreased in high grain (20 HG and 40 HG) compared to high forage diets (Table 5). Pentadecanoic (C_15:1_) and heptadecenoic acids (C_17:1_) were significantly (*p* < 0.001 and *p* < 0.01 respectively) decreased in the milk of high (40 HF and 40 HG) compared to low microalgae-fed goats, while the C_17:1_ was also decreased (*p* < 0.01) in high grain diets (Table 5). Palmitic (C_16:0_) and palmitoleic acids (C_16:1 n-7_) were significantly (*p* < 0.01 and *p* < 0.001 respectively) increased in high microalgae-fed goats (40 HF and 40 HG); while in palmitoleic acid, a significant interaction (*p* < 0.001) between sampling time and microalgae level was found (Table 5). Stearic (C_18:0_) and oleic acids (C_18:1 cis-9_) were significantly (*p* < 0.01) decreased by 40% and 23% in the milk of high (40 HF and 40 HG) compared to low microalgae-fed goats (Table 5). Total C_18:1 trans_ isomers (including VA) are significantly increased by 67% (*p* < 0.05) in high forage diets (20 HG and 40 HG) and by 46% in high microalgae-fed goats (40 HF and 40 HG) (Table 5). Linoleic acid (C_18:2 n-6 cis_) was significantly (*p* < 0.001) decreased in the milk of high microalgae-fed goats (40 HF and 40 HG), while linolenic acid (C_18:3 n-3_) was increased in high forage diets (20 HF and 40 HF; Table 5). Furthermore, a significant (*p* < 0.05) interaction was unveiled between microalgae level and F:C ratio in milk linoleic acid proportion (Table 5). Conjugated linoleic acid (CLA; C_18:2 cis-9, trans-11_) was significantly (*p* < 0.01) increased by 80% in high (40 HF and 40 HG) compared to low microalgae-fed goats (Table 5). The second major milk CLA isomers (CLA; C_18:2 trans-10, cis-12_) was significantly (*p* < 0.05) increased by 1.4-fold in high forage diets (20 HF and 40 HF) and by 84% (*p* < 0.05) in high microalgae-fed goats (40 HF and 40 HG; Table 5). Eicosatrienoic acid (C_20:3n3_) was significantly increased (*p* < 0.05) in high forage diets (20 HF and 40 HF) and high microalgae-fed goats (*p* < 0.001; 40 HF and 40 HG) as well (Table 5). Additionally, the eicosatrienoic acid was observed in a higher proportion on the 42nd experimental day, while all the tested interactions were observed significant (Table 5). Arachidonic acid (C_20:4 n-6_) was significantly (*p* < 0.001) increased by 2.3-fold in high microalgae-fed goats (40 HF and 40 HG) and the 42nd experimental day resulting in significant interactions between the tested factors (Table 5). Eicosapentaenoic (C_20:5 n-3_), nervonic (C_24:1 n-9_), and docosapentaenoic acid (DPA; C_22:5 n-6_) were significantly increased (*p* < 0.05) in high forage dietary treatments (20 HF and 40 HF), while DPA and nervonic acid were also increased by 71% (*p* < 0.001) and 1.1-fold (*p* < 0.001) in high (40 HF and 40 HG) compared to low microalgae fed goats (Table 5). In the case of nervonic acid, significant interactions (*p* < 0.01) were observed as well (Table 5). Docosahexaenoic acid (DHA; C_22:6 n-3_) was significantly (*p* < 0.001) increased by 56% in high microalgae-fed goats (40 HF and 40 HG) and as well as in the 42nd experimental day resulting in significant interactions amongst the investigated factors (Table 5).

The apparent transfer efficiency of DHA from feed to milk ranged between 23 and 20% in 20 HF and 20 HG-fed goats, respectively, while the increase of microalgae levels (40 g) considerably decreased its efficiency to 15% and 16% in 40 HF and 40 HG-fed goats, respectively. Then again, the apparent transfer efficiency of DPA ranged between 13% and 15% in 20 HF and 20 HG-fed goats respectively, while the increase of microalgae levels also suppressed its efficiency to 11% and 12 % in 40 HF and 40 HG diets, respectively.

**Figure 4:**
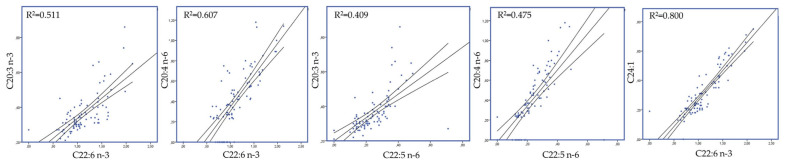
Pearson correlation of milk fatty acids of goats in four dietary treatments.

Figure 4 depicts the most significant correlations of milk fatty acids using a Pearson correlation. Eicosatrienoic acid (C_20:3n3_), arachidonic acid (C_20:4 n-6_), and nervonic (C_24:1 n-9_) acid were significantly (*p* < 0.01) positively correlated with DHA content in milk (*R*^2^ = 0.511, *R*^2^ = 0.607, and *R*^2^ = 0.800 respectively; Figure 4). The proportion of eicosatrienoic and arachidonic acid were also significantly (*p* < 0.01) positively correlated with DPA content in milk (*R*^2^ = 0.409 and *R*^2^ = 0.475 respectively; Figure 4).

### 3.4. Blood Plasma and Milk Oxidative Status

**Figure 5:**
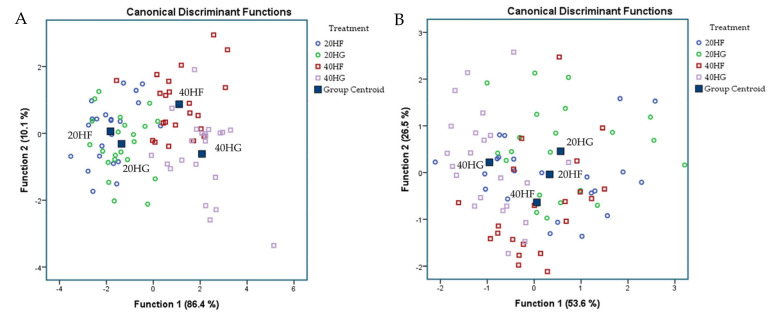
Discriminant plots separating (**A**) the four dietary treatments (20 HF; blue, 20 HG; green, 40 HF; red, and 40 HG; pink) according to pooled data of two sampling time (21st and 42nd experimental day) on the blood plasma oxidative status and (**B**) the four dietary treatments (20 HF; blue, 20 HG; green, 40 HF; red, and 40 HG; pink) according to pooled data of two sampling time (21st and 42nd experimental day) on the milk oxidative status. 20 HF: 20 g *Schizochytrium* spp. and high forage diet (60:40); 20 HG: 20 g *Schizochytrium* spp. and high grain diet (40:60); 40 HF: 40 g. *Schizochytrium* spp. and high forage diet (60:40); 40 HG: 40 g *Schizochytrium* spp. and high grain diet (40:60).

Figure 5A depicts a discriminant plot of blood plasma oxidative status of the four dietary treatments (20 HF; blue, 20 HG; green, 40 HF; red, and 40 HG; pink) throughout the experimental period. The proportions of the samples that were correctly classified were 71.6%. Wilks’ lambda was observed at 0.178 for Function 1 (*p* < 0.001) and 0.676 for Function 2 (*p* = 0.012), while the values of total antioxidant capacity using the FRAP method and the concentration of MDA were the variables that contributed the most based on a step wise method. The observation of blood oxidative status showed significant overlap, making it hard to conclude about a dependable classification. However, the centroids showed that the 20 HG and 20 HF groups were placed distanced from those of 40 HG and 40 HF. Figure 5B depicts the second discriminant plot of milk oxidative status of the four dietary treatments (20 HF; blue, 20 HG; green, 40 HF; red, and 40 HG; pink) throughout the experimental period. The proportions of the samples that were correctly classified were 52.3%. Wilks’ lambda was observed at 0.558 for Function 1 (*p* < 0.001) and 0.754 for Function 2 (*p* = 0.010), while the values of total antioxidant capacity using the ABTS method were the variable that contributed the most based on a step wise method. Despite the significance of the results (*p* < 0.05), the high Wilks’ lambda values and the severe overlapping of observations make it complicated to distinguish and classify samples amongst the four dietary groups

Table 6 presents the mean antioxidant enzyme activities, the total antioxidant capacity, and the oxidative status biomarkers of blood plasma and milk of goats fed with the four diets throughout the experimental period. The activity of catalase (CAT) in blood plasma was significantly (*p* < 0.05) decreased by 26% in high microalgae-fed goats (Table 6). Glutathione transferase (GSTs) and superoxide dismutase (SOD) activities in blood plasma were increased (*p* < 0.01 and *p* < 0.001 respectively) on the 42nd experimental day (Table 6). SOD activity was also increased (*p* < 0.001) in the blood plasma of high (40 HF and 40 HG) compared to low microalgae-fed goats (Table 6). These fluctuations in SOD activity resulted in significant (*p* < 0.001) interactions amongst the investigated factors (Table 6). Total antioxidant capacity measured by ABTS assay showed a significant decrease (*p* < 0.01) on the 42nd experimental day, while total antioxidant capacity measure by FRAP method found lower in high (40 HF and 40 HG) compared to low microalgae-fed goats (Table 6). The oxidative stress biomarkers were increased in high microalgae-fed goats indicating pro-oxidant incidence. More specifically, both protein carbonyls (CP) and malondialdehyde (MDA) concentration were increased (*p* < 0.001) in 40HF and 40 HG groups (*p* < 0.001). Additionally, MDA levels were found higher (*p* < 0.01) in high grain (20 HG and 40 HG) compared to high forage groups (Table 6).

Total antioxidant capacity of milk based on ABTS assay recorded lower in high microalgae-fed goats, while the CP was upsurged (*p* < 0.05) in the milk of goats consumed diets with a high level of grains (20 HG and 40 HG) compared to high forage groups (Table 6). Last but not least, both MDA and CP concentrations were reported higher on the 42nd experimental day (*p* < 0.01 and *p* < 0.001 respectively; Table 6).

## 4. Discussion

### 4.1. High Microalgae Level Decreased Feed Intake While F:C Ratio Remained Identical

The inclusion of high microalgae levels (40 HF and 40 HG) in goats’ diets resulted in a decreased feed intake of concentrates and consequently lower microalgae consumption. The decrease of concentrate intake may be attributed to microalgae’s fish-like flavor or the elevated fat content in the 40 HF and 40 HG diets (3.6% and 4.5% respectively) that may decrease the dry matter intake (DMI) due to cholecystokinin’s (hypophagic) effect on brain satiety centers [38]. On the other hand, wheat straw was decreased in high forage diets up to 50% due to its low palatability and animal resistance to long particles [39].

### 4.2. The Interaction between Fat-Rich Microalgae and the F:C Ratio Did Not Affect Milk Performance

It should be underlined here that since the two experimental phases took place at a different stage of lactation (approximately 84 vs. 140 days in milk) it was crucial to analyze the milk performance separately aiming to avoid such biases.

Although the manipulation of milk fat content by altering the F:C ratio is considered to be a well-justified strategy, shifting from 60:40 to 40:60 did not bring on considerable changes in our study. This lies in the simultaneous microalgae supplementation in the experimental diets that robustly regulate the fat secretion compared to the F:C ratio. More specifically, the marine origin fatty acids resulted in the most significant reduction of milk fat content compared to other animal and vegetable origin oils in a meta-analysis of 290 dietary treatments [14]. In this light, in our preliminary study, only the inclusion level of 40 g/day *Schizochytrium* spp. induced a severe milk fat depression in lactating goats compared to the normal fed [7] indicating that the PUFA supplementation level should be also considered providing design such dietary strategies.

### 4.3. High Microalgae and Concentrate Levels Improved Milk Fatty Acid Profile

Likewise, with the aforementioned hypotheses about milk fat content, the microalgae level was found to be the dominant factor of classifying blood and milk fatty acid profile compared to the F:C ratio. Palmitic acids were increased in both blood plasma and milk of goats consumed the high-microalgae level since palmitic acid appears to be a principal fatty acid in *Schizochytrium* spp. biomass [7]. Pendadecenoic (C_15:0_) formation is firmly dependent on rumen fermentation procedures. The reduction of C_15:0_ in high grain diets may be attributed to alterations in cellulolytic rumen microbes and their branched and odd-chain fatty acids formation [40].

Stearic acid constitutes the final product of the ruminal biohydrogenation process [41]. Reduction of stearic acid in both blood and milk of high (40 g) compared to low (20 g) microalgae-fed goats indicating that the inhibition of the biohydrogenation process depicted a dose-response. Interestingly, in our previous study, the escalated tested levels of 20, 40, and 60 g of *Schizochytrium* spp. in dairy goats’ diet did not demonstrate any tendency for dose-dependence using a moderate F:C ratio (50:50) [7]. These fluctuations may unveil the regulatory role of the F:C ratio even though no significant alterations were observed. Further to stearic acid, total trans C_18:1_ including the vaccenic acid which constitutes the precursor of CLA and were increased in the milk of high microalgae and high grain diets as a result of a partially inhibited biohydrogenation of dietary PUFA. In compliance with our findings, an increase in trans C_18:1_ isomers as a result of linolenic acid degradation products was found in high concentrate-fed ewes [42]. Similarly, a raise in the proportions of trans C_18:1_ isomers in goats’ milk was observed when the animals were fed a high grain diet supplemented with different oilseeds rich in PUFA (soybeans or canola seed) [43]. The CLA concentration was also increased in the milk of high microalgae-fed goats as a result of the increased abundance of its substrate in blood and milk (vaccenic acid). These observations were found to be of high importance with the aim of the design and implement sustainable and vegetable-friendly strategies to enrich dairy products with beneficial bio-lipids for consumer health. However, the reduction of stearic acid in the blood of high microalgae-fed goats due to the lower hydrogenation degree of PUFA decreased the availability of stearic acid in the mammary gland resulted in the suppression of the de novo synthesis of oleic acid through the activity of Δ9 desaturase [44].

The second most abundant CLA isomer in milk (C_18:2_ t10, c12) which has been related to the regulation of milk fat synthesis was increased in the milk of high microalgae-fed goats and high-grains diets as a result of a lower biohydrogenation activity of linoleic acid and its isomers within the rumen. In agreement with our findings, Thanh and Suksombat [45], observed higher levels of C_18:2_ t10, c12 in bovine milk of cows fed with high concentrate and PUFA-rich oilseeds diet. The concentration of both DPA and DHA fatty acids in blood and milk were found higher in high *Schizochytrium* spp. diets due to the elevated dietary intake. However, it is worth mentioning that their concentrations were recorded folds higher in the blood than that in the milk fat indicating their limited transfer efficiency as has been already reported [7,46]. This limitation in their transfer efficiency from feed to milk could be not only attributed to the biohydrogenation of PUFA within the rumen but also to the preference of these fatty acids to be incorporated into phospholipids and cholesteryl esters instead of triglycerides. The aforementioned preference makes these fatty acids unavailable for absorption by the mammary gland through lipoprotein lipase (LPL) compromising their efficiency [47].

The upsurge of docosadienoic acid (C_22:2 n-6_) in the blood of high microalgae-fed goats appears to be a well-established observation under DHA supplementation due to the biohydrogenation activity of *Butyrivibrio proteoclasticus* [48]. Eicosatrienoic acid (C_20:3 n-3_), arachidonic acid (C_20:4 n-6_), and nervonic (C_24:1 n-9_) were significantly increased in the milk of high-algae fed goats. The positive response of these fatty acids to dietary the *Schizochytrium* spp. triggered our interest since their dietary intake remained identical between treatments (there are not presented in *Schizochytrium* spp. biomass) while there is no known mechanism of their synthesis within the mammary gland. However, their positive correlation with DPA and DHA indicating that the aforementioned fatty acids have been formed as degradation products of DPA and DHA within the rumen. Although the length of carbon chain and abundance of double bonds are degraded during the PUFA degradation within the rumen, there is a high consistency regarding the location of the remaining double bonds. Thus, it is plausible to assume that eicosatrienoic acid constitutes the degradation product of DHA, while arachidonic acid has been formed by DPA. Interestingly, the formation of arachidonic acid in the milk of algae-fed ruminants has been reported previously as well [49,50].

The majority of medium-chain fatty acids (MCFA) with the principal the myristic and palmitic acid are de novo synthesized in the mammary gland. MCFA decline in high grain diets may be attributed to the inhibitory signaling of specific isomers in the lipogenic activity within the mammary gland [51]. More specifically, the proportion of C_18:2_ t10, c12, and C_18:1_ t10 which are increased in high-grain diets exert antilipogenic properties in the mammary gland [52].

From a human health point of view, the milk of high- compared to low- microalgae-fed goats portrayed an improved health-promoting and thrombogenic index, while the opposite pattern was found regarding the atherogenic index. It should be pointed out that the atherogenic index depicts a few limitations, hence the milk fatty acid profile should be holistically evaluated aiming to generate dependable and reliable results. More specifically, since the atherogenic index (AI) is strongly depended on MUFA and consequently on the principal milk MUFA e.g., oleic acid, the suppression of oleic acid as was explained above adversely affects AI. Taking into consideration the aforementioned, the combination of high microalgae (40) and grain diet (60:40) performed with the healthier milk composition from the human health point of view by enriching milk fat with DHA, DPA, nervonic acid, and altering the ω6/ω3 ratio toward a beneficial direction. DHA and DPA contribute to various aspects of human wellbeing, from the heart and vascular health to brain development and lifelong brain function. Indeed, these fatty acids participate in diverse processes including cell membrane structure, eicosanoid metabolism, gene transcription, and resolution of inflammation [53]. Additionally, nervonic acid is the core component of neural cells of the brain and neural tissue, which benefits brain health through improving the biosynthesis and maintenance of nerve cell myelin and also enhances neurodevelopment in premature infants. Nervonic acid can repair the damaged brain nerve pathways and promote the regeneration of nerve cells, which can be effective in the treatment of schizophrenia, psychosis, peroxisomal disorders, diabetes, alcoholism, and other conditions [54]. On the other hand, special attention should be given to arachidonic acid (ARA) enhancement since a few preliminary pieces of evidence are suggesting that ARA supplementation could increase platelet aggregation resulting in thrombotic incidences or upregulate a pro-inflammatory response through eicosanoid formation [55].

In agreement with our preliminary results [7], the upsurge of DPA and DHA intake decreases their apparent transfer efficiency from feed to milk. More specifically, the increase of microalgae levels from 20 to 40 g/day were negatively correlated with DPA and DHA transfer efficiency. On contrary, no differences were observed between F:C ratios. However, in this study considerably higher transfer efficiency was observed for DHA compared to our previous study and other reports [7,56]. More specifically, the apparent transfer efficiency of DHA was ranged between 23% to 20% in 20 HF and 20 HG diets respectively, while the increase of microalgae levels considerably decreased its efficiency to 15% and 16% in 40 HF and 40 HG diets respectively. With the exception of Keady et al. [57] who reported transfer efficiency values as high as 61% for EPA and 19–35% for DHA, it is generally thought that the transfer efficiency to the milk of EPA and DHA from fish oil added to the diet is rather low [58]. On the other hand, the apparent transfer efficiency of DPA was ranged between 13% to 15 % in 20 HF and 20 HG diets respectively, while the increase of algae levels suppressed its efficiency to 11% and 12 % in 40 HF and 40 HG diets respectively which come into agreement with our previous observations [7]. Discrepancies in DHA transfer efficiency between our experiments may be attributed to the lactation stage (from early to mid-lactation vs. mid to late-lactation), milk performance (higher milk yield in the present study), and experimental duration [58]. More specifically, the lower transfer efficiency of our preliminary study maybe lies in a bettered adaptation of the rumen microbiome during the 74 experimental days compared to 56 (14 as adaptation and 42 the main experimental period) of each experimental trial of the present study. The potential adaptation of rumen microbiota to *Schizochytrium* spp. inclusion may result in higher degradation of DPA and DHA during a longer interval experiment. However, considering the rumen microbiome of *Schizochytrium*-fed goats of our previous study, there was observed a significant alteration of the whole investigated species after the 20th experimental day in both species floated in the liquid or adhered to feed particles while no considerable changes were revealed between 40th and 60th day [10,11]. Nevertheless, the investigation of the genomic footprint of the ruminal microbial communities is not always corresponded to the microbes’ biochemical activity and metabolism and consequently their enzymatic potential [59]. Without ruling out the potential involvement of supplementation interval on DPA and DHA transfer efficiency, Wang et al. [60] reported that cows’ rumen microorganism activity is enhanced during the lactation stage resulting in a higher concentration of biohydrogenation intermediates. Thus, it could be assumed that the higher transfer efficiency of DHA in this study may lie in the earlier lactation stage.

### 4.4. The Oxidative Status Subverted the State of Affairs

PUFA appears to be prone to autoxidation and photooxidation [61]. Thus, their inclusion in animals’ diets conceals severe challenges regarding organism’s oxidative status. Whereas, specific saturated fatty acids such as palmitic acid could trigger cell defended mechanisms inducing a cascade of pro-oxidant incidence as well [62]. Both endogenous and exogenous mechanisms stand by cell viability by neutralizing the reactive oxygen species (ROS) [63]. In this context, the increase of SOD activity in the blood of high algae-fed goats could indicate the organism’s response to oxidative imbalances. More specifically, it has been reported that the supplementation of human diets with ω3 PUFA [64], or the inclusion of palmitic acid in rat diets [65] promoting the formation of superoxide anion (O_2_^•−^), through the mitochondria respiratory. The increased concentration of O_2_^•−^ may cause an increase in SOD activity aiming to neutralize it. Another important generator of the superoxide anion is the NADPH oxidase (NOX) [66]. In our previous study, the escalated levels of *Schizochytrium* spp. in goats’ diet increased the activity of plasma NOX in a dose-depended manner [16].

On the other hand, the high algae level in goats’ diet decreased the activity of CAT in blood. It could be hypothesized that the increased levels of superoxide anion combined with the upsurge activity of SOD resulted in the formation of hydrogen peroxide (H_2_O_2_). The formed hydrogen peroxide can cause an increase in the concentration of hydroxyl radical (OH^−^), as a consequence of the Fenton reaction [67] inhibiting the activity of CAT [68]. Furthermore, the F:C ratio affected the CAT activity in blood plasma as well. More specifically, the higher CAT activity in high grain diets could be lies in the higher availability of high-digestible starch sources due to grain content [69].

The rise of MDA levels which reflect the degree of lipid peroxidation could be attributed to the increased levels of PUFA in high microalgae diets [70]. The MDA is one of the main intermediates between lipid peroxidation and oxidative stress [31]. Interestingly, blood MDA was further increased in goats who consumed high grain diets compared to those fed with high forages. Blood MDA values were ranged between 1.23 and 1.94 μM indicating an increased grade of lipid peroxidation compared to previous studies on goats (0.41–1.55 μM) [16] and dairy sheep (0.60–0.89 μM) [71]. The mechanism that underlies this observation could be related to the high starch content of rumen which may increase the lipopolysaccharides levels in their ruminal fluid, resulting in a pro-inflammatory response and consequently to oxidative imbalances [17]. Another hypothesis could be lying on a robust vitamin E and A degradation in the rumen due to higher starch diets [18,19]. Notably, vitamin E appears to have a pivotal protective role against lipid peroxidation [72,73]. Moreover, protein carbonyls could be formed either directly through ROS action on the amino acid side chain [74], or indirectly via peroxyl radicals formed by lipid peroxidation [75]. Nevertheless, protein carbonyls are formed with much rapid grade due to the detrimental action of lipid peroxidation by-products rather than the ROS action per se [76].

Despite the presence of oxidative imbalances in the goats’ organism (blood) attributed both to higher microalgae level and high grains proportion, the milk’s oxidative stability portrayed an improved response to experimental diets since the principal indicator of oxidative status was not affected (MDA). However, total antioxidant capacity measured by ABTS presented low values in the milk of goats fed the high microalgae diets. Further to the microalgae inclusion level, the F:C ratio affected milk’s oxidative stability as well. Milk protein carbonyls were increased in high grain compared to a high forage diet. The exact mechanism of carbonyls formation in the milk of high grain diets while in blood plasma they were remained unaffected is still unclear. The only dependable assumption that could be given is related to the higher metal (Cu, Zn) concentration of milk compared to blood [77]. More specifically, the carbonylation of amino acid side chains constitutes a process that is commonly taking place as a result of the interaction between metals and ROS [78].

## 5. Conclusions

Although the inclusion of 40 g *Schizochytrium* spp. and high grain diet (40:60) showed the healthier milk fatty acid profile without adversely affect milk performance, diet palatability was slightly compromised and goat’s organism and milk oxidative balance were severely disturbed. On the other hand, the supplementation of 20 g *Schizochytrium* spp. combined with high forage diet (60:40) appears to be a well-justified strategy to enrich dairy products with essential biomolecules for human health without negative impact in its oxidative stability. The improved transfer efficiency of DHA during early lactation triggers further research to validate, such as if the farm-scale implementation should target the period of the early lactation for short intervals aiming to produce PUFA-rich dairy products with the optimum efficiency and most sustainable manner.

## Figures and Tables

**Table 1:** Ration components (Kg/goat/day) and chemical composition (g/day) of the diets were administered to the four groups (20 HF, 20 HG, 40 HF, and 40 HG) of goats involved in the trials.

	Treatment			
	20 HF	20 HG	40 HF	40 HG
Diet components (Kg per goat)				
Alfalfa hay	1.2	0.7	1.2	0.7
Wheat straw	0.3	0.18	0.3	0.18
Concentrate mix	1	1.3	1	1.3
*Schizochytrium* spp. (g)	20	20	40	40
Forage to Concentrate (F:C) ratio	1.5:1 (60:40)	0.88:1.3 (40:60)	1.5:1 (60:40)	0.88:1.3 (40:60)
Dry Matter	2282	1989	2298	2000
Ash	188	144	192	142
Crude Protein	312	311	312	311
Ether Extract	82.3	87.9	90.3	97.0
Ash-free NDF treated with amylase	932	712	931	710
Acid Detergent Fiber	608	399	605	409
Non Fibrous Carbohydrate	987	925	976	920
Starch	474	542	462	542
NDF/Starch	2.0	1.3	2.0	1.3

20 HF: 20 g *Schizochytrium* spp. and high forage diet (60:40); 20 HG: 20 g *Schizochytrium* spp. and high grain diet (40:60); 40 HF: 40 g *Schizochytrium* spp. and high forage diet (60:40); 40 HG: 40 g *Schizochytrium* spp. and high grain diet (40:60).

**Table 2:** Alfalfa hay, wheat straw and concentrates fatty acid profile (FA) (% of total FA).

Fatty Acid	Concentrates				Forages	
	20 HF	20 HG	40 HF	40 HG	Alfalfa Hay	Wheat Straw
Myristic acid (C_14:0_)	2.48	2.12	3.1	3.18	6.2	0
Palmitic acid (C_16:0_)	21.94	22.99	20.22	23.77	36.77	29.88
Stearic acid (C_18:0_)	1.92	2.06	1.55	1.87	2.33	4.86
Oleic acid (C_18:1 cis-9_)	28.95	31.83	22.13	27.08	2.49	34.77
Linoleic acid (C_18:2 n-6 cis_)	31.96	31.04	31.09	27.76	18.27	21.95
Eicosanoic acid (C_20:0_)	0.22	0.23	0.17	0.2	0.64	0.82
Linolenic acid (C_18:3 n-3_)	1.07	0.96	1.13	0.88	30.68	1.86
Eicosatrienoic acid (C_20:3n3_)	0.44	0.37	0.56	0.47	1.5	1.37
Lignoceric acid (C_24:0_)	0.32	0.25	0.26	0.25	0	0.73
Docosapentaenoic acid (C_22:5 n-6_)	2.42	1.92	4.7	3.78	0	0
Docosahexaenoic acid (C_22:6 n-3_)	6.71	5.25	13.76	10.21	0	0

20 HF: 20 g *Schizochytrium* spp. and high forage diet (60:40); 20 HG: 20 g *Schizochytrium* spp. and high grain diet (40:60); 40 HF: 40 g *Schizochytrium* spp. and high forage diet (60:40); 40 HG: 40 g *Schizochytrium* spp. and high grain diet (40:60).

**Table 3:** Feed intake on a fresh matter basis (Kg/goat and percentage of the consumed quantities compared to given) and nutrients consumption (g) of the four groups (20 HF, 20 HG, 40 HF, and 40 HG) of goats involved in the trials.

	Treatment			
	20 HF	20 HG	40 HF	40 HG
Diet Consumption				
Alfalfa hay	1.2 (100)	0.7 (100)	1.2 (100)	0.7 (100)
Wheat straw	0.2 (66)	0.18 (99)	0.15 (50)	0.16 (90)
Concentrate mix	0.97 (97)	1.29 (99)	0.84 (84)	1.09 (84)
*Schizochytrium* spp. g	19.3 (97)	19.8 (99)	33.7 (84)	33.2 (83)
*Schizochytrium* spp. % of DMI	0.89	1	1.68	1.86
Forage to Concentrate (F:C) ratio	1.4:0.97 (59:41)	0.88:1.29 (40:60)	1.35:0.84 (61:39)	0.76:1.09 (41:59)
Nutrients Intake				
Dry Matter	2161	1980	2010	1788
Ash	179	144	173	131
Crude Protein	305	309	286	276
Ether Extract	79	87	76	83
Ash-free NDF amylase treated	853	709	788	649
Acid Detergent Fiber	555	398	515	383
Non Fibrous Carbohydrate	954	920	866	810
Starch	460	538	393	459
NDF/Starch	1.9	1.3	2.0	1.4

20 HF: 20 g *Schizochytrium* spp. and high forage diet (60:40); 20 HG: 20 g *Schizochytrium* spp. and high grain diet (40:60); 40 HF: 40 g *Schizochytrium* spp. and high forage diet (60:40); 40 HG: 40 g *Schizochytrium* spp. and high grain diet (40:60).

**Table 4:** The mean individual fatty acids (FA) (% of total FA) in the blood plasma of goats fed diets (20 HF, 20 HG, 40 HF, and 40 HG) with different levels of *Schizochytrium* spp. (20 g and 40 g/goat/day) and two different forage to concentrate ratios (60:40 and 40:60) throughout the experimental period (21st and 42nd experimental days).

	Dietary Treatment (D)						Sampling Time (S)			Effect						
	Forage/Concentrate			Algae Level			Sampling Day			Effect			Interaction Effect			
	60/40	40/60	SEM ^a^	20 g	40 g	SEM ^a^	21	42	SEM ^a^	F/C	ALG	S	F/C × A	F/C × S	A × S	F/C × A × S
C_14:0_	0.432	0.460	0.033	0.292 ^a^	0.599 ^b^	0.057	0.344 ^a^	0.548 ^b^	0.052	NS	***	**	NS	NS	***	NS
C_16:0_	15.69	16.57	0.544	15.47 ^a^	16.79 ^b^	0.623	16.26	16.00	0.558	NS	*	NS	NS	**	NS	NS
C_16:1 n-7_	0.423	0.617	0.072	0.351 ^a^	0.689 ^b^	0.091	0.541	0.499	0.088	t	***	NS	NS	NS	NS	NS
C_17:0_	0.949	0.799	0.075	0.842	0.906	0.087	0.832	0.916	0.091	NS	NS	NS	NS	NS	NS	NS
C_18:0_	20.25	15.72	2.039	22.53 ^a^	13.45 ^b^	2.324	18.68	17.29	2.199	NS	***	NS	NS	NS	NS	NS
C_18:1 trans_	1.35	1.01	0.287	1.34	1.02	0.320	1.31	1.06	0.312	NS	NS	NS	NS	NS	NS	NS
C_18:1 trans-11_	4.40	7.56	1.152	3.73 ^a^	8.23 ^b^	1.342	5.42	6.54	1.224	t	***	t	*	NS	NS	NS
C_18:1 cis-9_	8.66	8.71	0.390	9.19 ^a^	8.18 ^b^	0.407	9.12 ^a^	8.25 ^b^	0.409	NS	***	***	NS	*	NS	*
C_18:2 n-6 trans_	0.527	0.479	0.070	0.478	0.528	0.076	0.533	0.472	0.082	NS	NS	NS	*	NS	*	NS
C_18:2 n-6 cis_	21.02	22.11	0.710	24.98 ^a^	18.15 ^b^	0.859	22.11	21.03	0.906	NS	***	NS	*	NS	**	*
C_18:3 n-3_	1.81 ^a^	1.01 ^b^	0.166	1.44	1.39	0.178	1.43	1.39	0.173	**	NS	NS	NS	NS	NS	NS
C_20:3 n-6_	0.424	0.544	0.071	0.279 ^a^	0.690 ^b^	0.092	0.416 ^a^	0.553 ^b^	0.079	NS	***	*	NS	NS	NS	NS
C_20:3 n-3_	8.63	9.06	0.333	7.43 ^a^	10.26 ^b^	0.414	8.61 ^a^	9.09 ^b^	0.354	NS	***	*	*	***	**	NS
C_22:2 n-6_	4.85	5.21	0.316	3.09 ^a^	6.98 ^b^	0.342	4.37 ^a^	5.69 ^b^	0.322	NS	***	***	*	*	NS	NS
C_22:5 n-6_	1.36	1.50	0.094	0.922 ^a^	1.94 ^b^	0.130	1.29 ^a^	1.57 ^b^	0.128	NS	***	*	NS	NS	NS	NS
C_22:6 n-3_	9.06	8.47	0.260	7.60 ^a^	9.93 ^b^	0.317	8.61	8.92	0.296	NS	***	NS	NS	NS	**	**

Means with different superscript letters (a, b) between forage to concentrate ratio, algae levels and sampling time differ significantly; * *p* < 0.05, ** *p* < 0.01, *** *p* < 0.001, t = trend *p* < 0.10. ^a^ SEM: Standard error of the means. 20 HF (*n* = 11 goats): 20 g *Schizochytrium* spp. and high forage diet (60:40); 20 HG (*n* = 11 goats): 20 g *Schizochytrium* spp. and high grain diet (40:60); 40 HF (*n* = 11 goats): 40 g *Schizochytrium* spp. and high forage diet (60:40); 40 HG (*n* = 11 goats): 40 g *Schizochytrium* spp. and high grain diet (40:60).

**Table 5:** The mean individual fatty acids (FA) (% of total FA), grouped FA, FA health indices, and Δ-9 desaturase indices in the milk of goats fed diets (20 HF, 20 HG, 40 HF, and 40 HG) with different levels of *Schizochytrium* spp. (20 g and 40 g/goat/day) and two different forage to concentrate ratios (60:40 and 40:60) throughout the experimental period (21st and 42nd experimental days).

	Dietary Treatment (D)						Sampling Time (S)			Effect						
	Forage/Concentrate			Algae Level			Sampling Day			Effect			Interaction Effect			
	60/40	40/60	SEM ^a^	20 g	40 g	SEM ^a^	21	42	SEM ^a^	F/C	ALG	S	F/C × A	F/C × S	A × S	F/C × A × S
C_4:0_	2.83	2.73	0.094	2.79	2.76	0.101	2.75	2.80	0.106	NS	NS	NS	NS	NS	NS	NS
C_6:0_	3.25	3.58	0.268	3.21	3.63	0.291	3.55	3.28	0.300	NS	NS	NS	NS	NS	NS	NS
C_8:0_	3.75	3.84	0.135	3.77	3.82	0.148	3.82	3.78	0.140	NS	NS	NS	NS	*	NS	NS
C_10:0_	11.71	11.77	0.367	11.73	11.75	0.445	12.04 ^a^	11.44 ^b^	0.393	NS	NS	**	NS	NS	NS	NS
C_11:0_	0.171	0.108	0.025	0.152	0.128	0.027	0.146	0.133	0.026	NS	NS	NS	NS	NS	*	NS
C_12:0_	4.64	4.56	0.250	4.65	4.54	0.289	4.78	4.41	0.259	NS	NS	***	NS	NS	NS	NS
C_13:0_	0.029	0.014	0.011	0.030 ^a^	0.014 ^b^	0.012	0.026	0.018	0.013	NS	*	NS	*	NS	NS	NS
C_14:0_	10.26	9.67	0.304	9.97	9.95	0.335	10.08	9.85	0.318	NS	NS	NS	NS	NS	NS	NS
C_14:1_	0.304	0.275	0.015	0.293	0.287	0.017	0.304 ^a^	0.277 ^b^	0.017	NS	NS	*	NS	NS	NS	NS
C_15:0_	0.969 ^a^	0.767 ^b^	0.037	0.853	0.883	0.041	0.888 ^a^	0.848 ^b^	0.037	***	NS	***	NS	NS	*	NS
C_15:1_	0.227	0.208	0.022	0.257 ^a^	0.178 ^b^	0.026	0.232 ^a^	0.203 ^b^	0.024	NS	***	*	NS	NS	NS	NS
C_16:0_	29.19	27.99	0.670	27.89 ^a^	29.29 ^b^	0.730	28.57	28.61	0.693	NS	**	NS	NS	NS	NS	NS
C_16:1 n-7_	0.497	0.455	0.023	0.405 ^a^	0.547 ^b^	0.028	0.403 ^a^	0.549 ^b^	0.029	NS	***	***	NS	NS	***	NS
C_17:1_	0.097 ^a^	0.028 ^b^	0.013	0.090 ^a^	0.034 ^b^	0.018	0.078 ^a^	0.046 ^b^	0.015	**	**	*	NS	NS	NS	NS
C_18:0_	7.07	5.67	0.912	7.98 ^a^	4.76 ^b^	1.100	6.33	6.41	0.952	NS	**	NS	NS	NS	NS	NS
C_18:1 trans_	5.92 ^a^	9.94 ^b^	1.189	6.45 ^a^	9.42 ^b^	1.364	7.66	8.20	1.219	*	**	NS	NS	NS	*	NS
C_18:1 cis-9_	12.52	11.36	0.747	13.48 ^a^	10.40 ^b^	0.931	11.86	12.03	0.784	NS	**	NS	NS	NS	NS	NS
C_18:2 n-6 trans_	0.410	0.371	0.031	0.407	0.375	0.036	0.400	0.382	0.032	NS	NS	NS	NS	NS	NS	***
C_18:2 n-6 cis_	2.03	2.24	0.088	2.39 ^a^	1.88 ^b^	0.101	2.080	2.193	0.109	NS	***	NS	*	NS	NS	NS
C_18:3 n-3_	0.443 ^a^	0.250 ^b^	0.034	0.331	0.361	0.039	0.347	0.346	0.035	***	NS	NS	NS	NS	NS	NS
C_20:0_	0.122	0.109	0.006	0.124 ^a^	0.107 ^b^	0.008	0.114	0.116	0.007	NS	*	NS	NS	NS	**	NS
C_18:2 cis-9,trans-11_	1.21	1.26	0.175	0.879 ^a^	1.59 ^b^	0.219	1.16	1.31	0.184	NS	**	NS	NS	NS	NS	NS
C_18:2 trans-10, cis-12_	0.037 ^a^	0.089 ^b^	0.017	0.044 ^a^	0.081 ^b^	0.021	0.069	0.057	0.019	*	*	NS	NS	NS	*	NS
C_22:0_	0.052	0.029	0.012	0.059 ^a^	0.023 ^b^	0.016	0.052 ^a^	0.030 ^b^	0.013	NS	*	*	NS	NS	NS	NS
C2_0:3 n-3_	0.320 ^a^	0.393 ^b^	0.021	0.302 ^a^	0.410 ^b^	0.021	0.339 ^a^	0.374 ^b^	0.021	*	***	***	***	***	***	***
C_20:4 n-6_	0.380	0.450	0.036	0.190 ^a^	0.641 ^b^	0.039	0.328 ^a^	0.502 ^b^	0.039	NS	***	***	**	NS	**	*
C_20:5 n-3_	0.009 ^a^	0.054 ^b^	0.013	0.025	0.038	0.015	0.043 ^a^	0.020 ^b^	0.014	*	NS	*	NS	NS	NS	NS
C_24:1 n-9_	0.283 ^a^	0.357 ^b^	0.018	0.203 ^a^	0.437 ^b^	0.021	0.292 ^a^	0.348 ^b^	0.019	**	***	***	**	**	***	***
C_22:5 n-6_	0.215 ^a^	0.263 ^b^	0.016	0.176 ^a^	0.302 ^b^	0.019	0.213 ^a^	0.265 ^b^	0.019	*	***	**	NS	*	NS	NS
C_22:6 n-3_	1.05	1.17	0.055	0.865 ^a^	1.35 ^b^	0.059	1.05 ^a^	1.17 ^b^	0.060	NS	***	**	***	*	***	**
Grouped Fatty Acids																
SCFA	21.71	22.03	0.661	21.65	22.09	0.740	22.30 ^a^	21.44 ^b^	0.691	NS	NS	*	NS	NS	NS	NS
ΜCFA	45.08	42.99	0.741	43.39	44.68	0.881	44.35	43.73	0.789	t	t	NS	NS	NS	NS	NS
LCFA	7.25	5.81	0.924	8.17 ^a^	4.89 ^b^	1.116	6.50	6.56	0.965	NS	**	NS	NS	NS	NS	NS
ΜUFA	19.86 ^a^	22.63 ^b^	0.833	21.18	21.30	0.923	20.83 ^a^	21.65 ^b^	0.871	*	NS	*	NS	NS	NS	NS
PUFA	5.69	6.17	0.205	5.21 ^a^	6.65 ^b^	0.235	5.62 ^a^	6.23 ^b^	0.245	NS	***	**	*	NS	**	**
SFA	74.04 ^a^	70.83 ^b^	0.959	73.21 ^a^	71.67 ^b^	1.047	73.15 ^a^	71.73 ^b^	1.029	*	*	*	NS	NS	NS	NS
UFA	25.55 ^a^	28.79 ^b^	0.965	26.38 ^a^	27.96 ^b^	1.054	26.45 ^a^	27.89 ^b^	1.035	*	*	*	NS	NS	NS	NS
SFA/UFA	2.99 ^a^	2.52 ^b^	0.139	2.84 ^a^	2.67 ^b^	0.150	2.84 ^a^	2.67 ^b^	0.147	*	*	*	*	NS	NS	NS
ω6	3.07 ^a^	3.41 ^b^	0.113	3.21	3.28	0.127	3.09 ^a^	3.40 ^b^	0.134	*	NS	**	NS	NS	NS	NS
ω3	1.82	1.87	0.074	1.52 ^a^	2.16 ^b^	0.079	1.78 ^a^	1.91 ^b^	0.079	NS	***	**	***	*	***	**
ω6/ω3	1.72	2.05	0.129	2.23 ^a^	1.54 ^b^	0.144	1.79	1.98	0.144	NS	***	NS	*	NS	NS	NS
Fatty Acids Health Indices																
AI	2.37 ^a^	1.79 ^b^	0.171	1.41 ^a^	2.74 ^b^	0.188	1.46 ^a^	2.69 ^b^	0.184	*	***	***	NS	NS	***	*
ΤΙ	1.55	1.49	0.032	1.62 ^a^	1.41 ^b^	0.039	1.52	1.51	0.034	NS	***	NS	*	NS	**	NS
HΡΙ	0.669	0.679	0.006	0.666 ^a^	0.683 ^b^	0.007	0.670 ^a^	0.679 ^b^	0.007	NS	***	*	NS	NS	*	NS
Δ_−9_ Desaturase Indices																
C_14:1_/C_14:0_	0.030	0.029	0.002	0.030	0.029	0.002	0.030	0.028	0.002	NS	NS	NS	NS	NS	NS	NS
C_16:1_/C_16:0_	0.017	0.016	0.001	0.015 ^a^	0.019 ^b^	0.001	0.014 ^a^	0.019 ^b^	0.001	NS	***	***	NS	NS	***	NS
C_18:1_/C_18:0_	2.29	2.81	0.250	2.11 ^a^	2.99 ^b^	0.313	2.60	2.50	0.261	NS	**	NS	NS	NS	NS	NS

Means with different superscript letters (a, b) between forage to concentrate ratio, algae levels and sampling time differ significantly; * *p* < 0.05, ** *p* < 0.01, *** *p* < 0.001, t = trend *p* < 0.10. ^a^ SEM: Standard error of the means. 20 HF (*n* = 11 goats): 20 g *Schizochytrium* spp. and high forage diet (60:40); 20 HG (*n* = 11 goats): 20 g *Schizochytrium* spp. and high grain diet (40:60); 40 HF (*n* = 11 goats): 40 g *Schizochytrium* spp. and high forage diet (60:40); 40 HG (*n* = 11 goats): 40 g *Schizochytrium* spp. and high grain diet (40:60).

**Table 6:** Enzyme activities (Units/mL), total antioxidant capacity, and oxidative status biomarkers in blood plasma and milk of goats fed diets (20 HF, 20 HG, 40 HF, and 40 HG) with different levels of *Schizochytrium* spp. (20 g and 40 g/goat/day) and two different forage to concentrate ratios (60:40 and 40:60) throughout the experimental period (21st and 42nd experimental days).

	Dietary Treatment (D)						Sampling Time (S)			Effect						
	Forage/Concentrate			Algae Level			Sampling Day			Effect			Interaction Effect			
	60/40	40/60	SEM ^a^	20 g	40 g	SEM ^a^	21	42	SEM ^a^	F/C	ALG	S	F/C × A	F/C × S	A × S	F/C × A × S
Blood Plasma																
CAT units/mL	5.54	7.38	0.841	7.39 ^a^	5.53 ^b^	0.979	6.01	6.91	0.937	0.065	*	NS	NS	***	NS	NS
GSH-Px units/mL	0.103	0.107	0.005	0.106	0.104	0.006	0.102	0.108	0.005	NS	NS	0.063	NS	***	NS	NS
GR units/mL	0.065	0.059	0.004	0.061	0.063	0.005	0.060	0.065	0.004	NS	NS	NS	NS	NS	NS	NS
GSTs units/mL	0.248	0.266	0.012	0.246	0.268	0.016	0.241 ^a^	0.273 ^b^	0.013	NS	NS	**	NS	NS	NS	NS
SOD units/mL	13.65	14.37	0.314	12.96 ^a^	15.06 ^b^	0.368	13.16 ^a^	14.85 ^b^	0.341	NS	***	***	NS	NS	NS	***
ABTS %	40.88	40.14	0.492	39.98	41.04	0.670	41.41 ^a^	39.61 ^b^	0.631	NS	NS	**	**	NS	***	NS
FRAP μΜ	0.984	1.11	0.053	1.14 ^a^	0.955 ^b^	0.070	1.02	1.07	0.063	NS	*	NS	NS	NS	NS	**
CP nmol/ml	3.74	3.89	0.104	3.42 ^a^	4.21 ^b^	0.134	3.50 ^a^	4.13 ^b^	0.146	NS	***	***	NS	NS	NS	NS
MDA μΜ	1.33 ^a^	1.84 ^b^	0.119	1.23 ^a^	1.94 ^b^	0.137	1.57	1.60	0.127	**	***	NS	NS	NS	NS	NS
Milk																
LPO units/mL	0.804	0.737	0.042	0.768	0.772	0.044	0.765	0.775	0.046	NS	NS	NS	NS	NS	NS	NS
SOD units/mL	33.28	31.96	1.473	32.01	33.22	1.703	36.39 ^a^	28.85 ^b^	1.959	NS	NS	***	**	NS	NS	*
ABTS %	13.59	13.98	0.477	15.56 ^a^	12.00 ^b^	0.615	14.27	13.29	0.676	NS	***	NS	*	*	***	*
FRAP μΜ	1.44	1.39	0.062	1.46	1.37	0.073	1.65 ^a^	1.18 ^b^	0.078	NS	NS	***	*	NS	***	**
CP nmol/mL	2.87 ^a^	3.29 ^b^	0.119	2.99	3.16	0.153	2.91 ^a^	3.24 ^b^	0.142	*	NS	**	NS	NS	**	***
MDA μΜ	0.597	0.603	0.049	0.600	0.600	0.056	0.421 ^a^	0.779 ^b^	0.053	NS	NS	***	**	NS	NS	*

Means with different superscript letters (a, b) between forage to concentrate ratio, algae levels and sampling time differ significantly; * *p* < 0.05, ** *p* < 0.01, *** *p* < 0.001. ^a^ SEM: Standard error of the means. 20 HF (*n* = 11 goats): 20 g *Schizochytrium* spp. and high forage diet (60:40); 20 HG (*n* = 11 goats): 20 g *Schizochytrium* spp. and high grain diet (40:60); 40 HF (*n* = 11 goats): 40 g *Schizochytrium* spp. and high forage diet (60:40); 40 HG (*n* = 11 goats): 40 g *Schizochytrium* spp. and high grain diet (40:60).

## Data Availability

Data are contained within the article and Supplementary Materials.

## Supplementary Materials

The following are available online at https://www.mdpi.com/article/10.3390/foods10061322/s1, Table S1. Ingredients of concentrate (g/Kg) of the four diets, Table S2. Feed chemical composition (%), Figure S1. Average body weight (Kg) of the four groups (20 HF, 20 HG, 40 HF, and 40 HG) of goats involved in the trial, Table S3. Average body weight (Kg) of the 20 HF and 20 HG-fed goats throughout the experimental phase, Table S4. Average body weight (Kg) of the 40 HF and 40 HG-fed goats throughout the experimental phase, Table S5. Spearman correlation between blood plasma fatty acids and milk.
