# Supplementary material for: Effects of Inclusion of Schizochytrium spp. and Forage-to-Concentrate Ratios on Goats’ Milk Quality and Oxidative Status
Source: Foods. 2021 Jun 8;10(6):1322. doi: 10.3390/foods10061322 (PMC8228103; doi:10.3390/foods10061322)
Supplement: Supplementary file 1 [file foods-10-01322-s001.zip › foods-1248247-supplementary.pdf]

Table S1. Ingredients of concentrate (g/Kg) of the four diets.

	Concentrates			
	**20HF**	**20HG**	**40HF**	**40HG**
Maize grain	387	331.6	357	326.3
Barley grain	200	200	200	200
Wheat middlings	210	210	210	210
Sunflower meal,	80	80	80	80
Soybean meal	60	120	70	110
Calcium phosphate	15	15	15	15
Calcium carbonate	5	5	5	5
Salt	3	3	3	3
Mineral and vitamin	20	20	20	20
Schizochytrium spp. (g)	20	15.4	40	30.7

20HF: 20g *Schizochytrium spp* and high forage diet (60:40); 20HG: 20g *Schizochytrium spp* and high grain diet (40:60); 40HF: 40g *Schizochytrium spp* and high forage diet (60:40); 40HG: 40g *Schizochytrium spp* and high grain diet (40:60)

Table S2. Feed chemical composition (%).

	Concentrates				Forages	
	**20HF**	**20HG**	**40HF**	**40HG**	**Alfalfa hay**	**Wheat straw**
Dry Matter	90.8	91.0	92.4	91.8	91.1	93.7
Ash	4.1	4.5	4.5	4.3	10.3	7.7
Crude Protein	13.6	16	13.6	16	14	2.5
Ether Extract	6.1	5.8	6.9	6.5	1.3	1.9
Ash-free NDF treated with amylase	21.9	22.5	21.8	22.4	41.5	71.5
Acid Detergent Fiber	7.2	8.9	8.0	8.6	30.6	50.7
Non Fibrous Carbohydrate	54.3	51.2	53.2	50.8	32.9	16.4
Starch	43.1	39.8	41.9	39.8	3.3	1.0

20HF: 20g *Schizochytrium spp* and high forage diet (60:40); 20HG: 20g *Schizochytrium spp* and high grain diet (40:60); 40HF: 40g *Schizochytrium spp* and high forage diet (60:40); 40HG: 40g *Schizochytrium spp* and high grain diet (40:60)

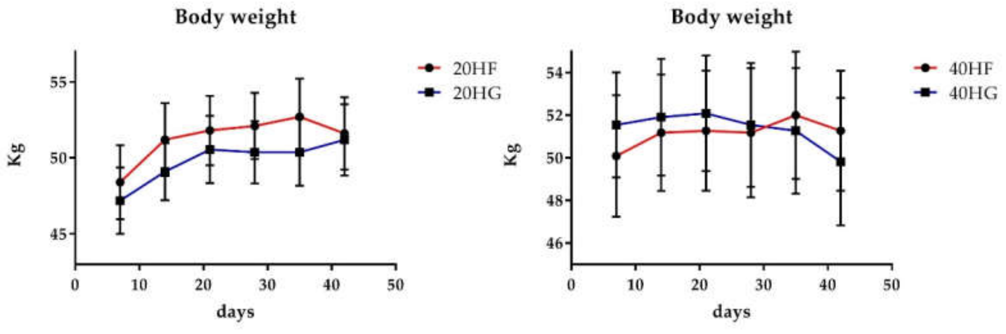

Figure S1. Average body weight (Kg) of the four groups (20HF, 20HG, 40HF, and 40HG) of goats involved in the trial. Error bars represent the Standard error of the means (SEM).
20HF: 20g *Schizochytrium spp* and high forage diet (60:40); 20HG: 20g *Schizochytrium spp* and high grain diet (40:60); 40HF: 40g *Schizochytrium spp* and high forage diet (60:40); 40HG: 40g *Schizochytrium spp* and high grain diet (40:60)

Table S3. Average body weight (Kg) of the 20HF and 20HG-fed goats throughout the experimental phase.

	Dietary treatment (D)			Sampling time (S)							Effect		
	20HF	*20HG*	*SEM*[a]	7	14	21	28	35	42	*SEM*[a]	D	S	DxS
Body Weight (Kg)	51.3	49.79	2.21	47.79^{a}	50.15^{b}	51.17^{c}	51.23^{c}	51.53^{c}	51.39^{c}	1.59	NS	***	NS

Means with different superscript letters (a, b) between dietary treatments and (a, b, and c) between sampling time differ significantly; * $p < 0.05$, ** $p < 0.01$, *** $p < 0.001$.

[a] SEM: Standard error of the means.

20HF: 20g Schizochytrium spp and high forage diet (60:40); 20HG: 20g Schizochytrium spp and high grain diet (40:60).

Table S4. Average body weight (Kg) of the 40HF and 40HG-fed goats throughout the experimental phase.

	Dietary treatment (D)			Sampling time (S)							Effect		
	40HF	*40HG*	*SEM*[a]	7	14	21	28	35	42	*SEM*[a]	D	S	DxS
Body Weight (Kg)	51.2	51.36	2.82	50.82^{ac}	51.55^{b}	51.68^{b}	51.36^{ab}	51.64^{ab}	50.55^{c}	2.01	NS	***	NS

Means with different superscript letters (a, b) between dietary treatments and (a, b, and c) between sampling time differ significantly; * $p < 0.05$, ** $p < 0.01$, *** $p < 0.001$.

[a] SEM: Standard error of the means.

40HF: 40g Schizochytrium spp and high forage diet (60:40); 40HG: 40g Schizochytrium spp and high grain diet (40:60)

Table S5. Spearman correlation between blood plasma fatty acids and milk.

Fatty acids on milk	Fatty acids on blood plasma																	
	$C_{14:0}$	$C_{15:0}$	$C_{16:0}$	$C_{16:1}$	$C_{17:0}$	$C_{18:0}$	$C_{18:1\ \textit{trans}}$	$C_{18:1\ \textit{trans}\text{-}11}$	$C_{18:1\ cis\text{-}9}$	$C_{18:2\ n\text{-}6\ trans}$	$C_{18:2\ n\text{-}6\ cis}$	$C_{18:3\ n\text{-}3}$	$C_{20:3\ n\text{-}6}$	$C_{20:0}$	$C_{20:3\ n\text{-}3}$	$C_{22:2\ n\text{-}6}$	$C_{22:5\ n\text{-}6}$	$C_{22:6\ n\text{-}3}$
$C_{4:0}$	-,063	,124	,048	,039	,152	-,047	,170	,053	,062	,155	,061	-,006	,094	,035	-,024	-,036	-,053	-,057
$C_{6:0}$	,009	,064	-,095	-,046	,106	,004	,003	-,082	-,108	,265*	-,018	-,002	,209	,242*	,095	,130	,081	,088
$C_{8:0}$	-,077	-,002	-,199	-,212	,038	,150	-,017	-,257*	-,062	,134	,035	,025	,021	,068	-,026	-,042	-,073	,025
$C_{10:0}$	-,081	,043	-,254*	-,255*	,025	,276*	-,129	-,338**	-,040	,128	,088	,135	-,119	-,052	-,113	-,132	-,135	-,029
$C_{11:0}$	-,099	,008	-,272*	-,110	,246*	,274*	,031	-,303**	-,163	,154	,015	,311**	,007	-,055	-,015	-,017	-,154	-,045
$C_{12:0}$	-,042	,023	-,134	-,220*	,092	,237*	-,029	-,259*	,147	,047	,064	,169	-,179	-,143	-,121	-,201	-,111	-,013
$C_{13:0}$	-,093	,139	-,054	-,077	,197	,099	,055	-,111	,097	-,130	,143	,088	-,192	-,241*	-,096	-,144	-,268*	-,135
$C_{14:0}$	,017	,146	-,081	-,082	,036	,159	-,077	-,152	,128	,106	,046	,191	-,095	-,147	-,104	-,182	-,033	,007
$C_{14:1}$	-,051	,148	,052	-,071	,169	,063	,143	-,042	,275*	-,144	,045	,083	-,088	-,185	-,113	-,162	-,057	-,110
$C_{15:0}$	,002	,093	,107	-,002	,059	-,004	,228*	,046	,177	-,118	-,096	,091	-,051	-,175	,091	,062	,011	,024
$C_{15:1}$	-,108	,203	-,065	-,110	,261*	,248*	-,001	-,137	,342**	,016	,182	,245*	-,351**	-,435**	-,381**	-,439**	-,246*	-,200
$C_{16:0}$	-,045	,066	,028	,013	-,291**	-,106	-,046	,061	-,226*	-,106	-,121	-,183	,124	,199	,148	,187	,100	,110
$C_{16:1\ n\text{-}7}$	,440**	-,130	,198	,225*	-,255*	-,334**	,235*	,258*	-,068	,055	-,358**	-,157	,371**	,265*	,489**	,516**	,381**	,476**
$C_{17:1}$	-,269*	,223*	-,228*	-,250*	,166	,308**	-,034	-,227*	,108	-,010	,185	,217	-,258*	-,370**	-,214	-,256*	-,345**	-,282*
$C_{18:0}$	,040	,083	,046	-,055	,124	,059	,052	,008	,069	-,138	,217	,173	-,202	-,252*	-,196	-,207	-,177	-,320**
$C_{18:1\ \textit{trans}}$	-,005	-,080	,061	,050	-,169	-,149	-,016	,118	,017	,080	-,169	-,288**	,184	,251*	,137	,144	,143	,213
$C_{18:1\ cis\text{-}9}$	,006	,051	-,025	-,018	,192	,129	,069	-,038	,109	-,088	,193	,211	-,223*	-,293**	-,235*	-,224*	-,215	-,288**
$C_{18:2\ n\text{-}6\ trans}$	-,053	,140	,076	,041	,136	,059	,104	,012	,258*	,013	-,070	,156	-,114	-,208	-,021	-,108	-,063	-,052
$C_{18:2\ n\text{-}6\ cis}$	-,125	-,029	-,096	-,074	,245*	,220*	-,003	-,077	,205	,089	,323**	,163	-,159	-,312**	-,341**	-,369**	-,327**	-,360**
$C_{20:0}$	-,023	,050	,050	-,012	,175	,058	,112	,036	,053	-,049	,209	,184	-,186	-,329**	-,232*	-,225*	-,175	-,309**
$C_{18:3\ n\text{-}3}$	,064	,172	,198	,097	,166	-,128	,230*	,168	,169	-,215	-,128	,004	,043	-,114	,174	,110	,019	-,053
$C_{18:2\ cis\text{-}9,trans\text{-}11}$	,036	-,099	,108	,072	-,282*	-,201	,032	,112	,040	-,054	-,313**	-,312**	,215	,303**	,341**	,293**	,221*	,416**
$C_{18:2\ trans\text{-}10,\ cis\text{-}12}$	,065	-,127	,029	,048	-,087	-,152	-,059	,096	-,038	,157	-,077	-,166	,215	,280*	,111	,196	,219*	,206
$C_{22:0}$	-,188	,214	-,007	-,059	,251*	,068	-,026	,019	-,026	-,135	,223*	,112	-,147	-,289**	-,121	-,218*	-,197	-,274*
$C_{20:3\ n\text{-}3}$	,104	-,131	,341**	,194	-,167	-,402**	,149	,375**	,089	-,265*	-,458**	-,305**	,254*	,408**	,498**	,450**	,432**	,574**
$C_{20:4\ n\text{-}6}$	,475**	-,155	,465**	,480**	-,157	-,644**	,212	,552**	-,154	-,205	-,694**	-,375**	,535**	,560**	,740**	,752**	,693**	,682**
$C_{20:5\ n\text{-}3}$	,063	-,080	-,182	-,020	-,004	,027	-,061	-,060	-,192	,226*	-,043	,064	,061	,059	,113	,096	,069	,187
$C_{24:1\ n\text{-}9}$	,348**	-,143	,424**	,458**	-,187	-,608**	,158	,546**	-,118	-,093	-,616**	-,427**	,570**	,566**	,620**	,622**	,675**	,623**
$C_{22:5\ n\text{-}6}$	,401**	-,119	,480**	,465**	-,147	-,608**	,232*	,560**	-,099	-,165	-,588**	-,340**	,454**	,534**	,627**	,643**	,616**	,599**
$C_{22:6\ n\text{-}3}$	,348**	-,079	,496**	,408**	-,103	-,610**	,203	,593**	,033	-,244*	-,546**	-,399**	,509**	,517**	,560**	,531**	,608**	,478**

**SCFA**	-,099	,028	-,208	-,193	,114	,187	-,103	-,264*	-,059	,205	,018	,062	,042	,070	-,009	-,020	-,058	-,001
**MCFA**	-,061	,107	-,092	-,060	-,173	,067	-,091	-,107	-,118	,025	-,067	,027	,002	,027	,055	,047	,023	,076
**LCFA**	,035	,085	,048	-,055	,132	,059	,053	,010	,072	-,138	,218*	,170	-,199	-,258*	-,197	-,212	-,181	-,326**
**MUFA**	,034	-,164	,080	,048	-,083	-,115	,058	,116	,087	-,001	-,058	-,210	,048	,074	-,005	,020	,062	,128
**PUFA**	,264*	-,161	,331**	,256*	-,121	-,441**	,190	,392**	,070	-,143	-,436**	-,344**	,422**	,435**	,510**	,467**	,433**	,507**
**SFA**	-,094	,176	-,162	-,115	,084	,220*	-,113	-,222*	-,086	,045	,174	,266*	-,164	-,178	-,139	-,154	-,176	-,244*
**UFA**	,096	-,176	,158	,112	-,089	-,218*	,104	,219*	,077	-,044	-,170	-,268*	,169	,186	,142	,157	,175	,244*
**SFA/UFA**	-,096	,176	-,156	-,112	,087	,217*	-,102	-,219*	-,076	,044	,172	,264*	-,168	-,184	-,141	-,157	-,176	-,246*
**AI**	-,068	,183	-,124	-,097	,035	,186	-,109	-,186	-,062	,033	,096	,226*	-,124	-,136	-,087	-,112	-,118	-,142
**$C_{14:1}$/ $C_{14:0}$**	-,038	,050	,072	-,021	,104	-,012	,141	,015	,217	-,175	,070	-,025	-,058	-,125	-,099	-,099	-,026	-,102
**$C_{16:1}$/ $C_{16:0}$**	,467**	-,140	,188	,189	-,177	-,281*	,240*	,218*	-,006	,052	-,298**	-,107	,318**	,211	,411**	,435**	,343**	,406**
**$C_{18:1}$/ $C_{18:0}$**	-,052	-,132	-,059	,080	-,115	-,050	-,011	-,003	-,061	,143	-,218*	-,151	,191	,213	,209	,235*	,167	,333**
**ω6**	,193	-,110	,242*	,209	,203	-,235*	,165	,315**	,130	-,074	-,173	-,074	,252*	,118	,183	,148	,182	,114
**ω3**	,339**	,007	,484**	,387**	-,010	-,589**	,278*	,580**	,031	-,273*	-,585**	-,321**	,477**	,445**	,608**	,559**	,574**	,509**
**ω6/ω3**	-,201	-,078	-,324**	-,230*	,115	,441**	-,145	-,367**	,063	,273*	,506**	,250*	-,333**	-,449**	-,518**	-,472**	-,500**	-,485**
**TI**	-,151	,101	-,183	-,175	,099	,301**	-,073	-,237*	,003	-,021	,462**	,268*	-,365**	-,418**	-,450**	-,425**	-,402**	-,506**
**HPI**	,050	-,158	,219*	,183	-,125	-,329**	,131	,301**	-,140	-,166	-,202	-,290**	,268*	,295**	,311**	,359**	,202	,133

* $p < 0.01$, ** $p < 0.001$
